# Socioeconomic disparities and life's essential 8: a focused review and considerations for improving cardiovascular health in the United States

**DOI:** 10.3389/fcvm.2026.1838136

**Published:** 2026-07-15

**Authors:** Vikas Sunder, Jennifer Garrow, Erik Van Iterson, Kardie Tobb, Nichole Opet, Sandra Tsai, Emelia Asamoah, Parveen Garg, Aniruddha Singh, Juliana Namutebi, Oby Ibe, Mansi Patil, Traci Mitchell, Keith Ferdinand

**Affiliations:** 1 Cleveland Clinic Heart, Vascular and Thoracic Institute, Cleveland, OH, United States; 2 Scripps Clinic, La Jolla, CA, United States; 3 Cone Health HeartCare, Greensboro, NC, United States; 4 Cleveland Clinic Digestive Disease and Surgery Institute, Cleveland, OH, United States; 5 Stanford University School of Medicine, Palo Alto, CA, United States; 6 Nursing Department, Morgan State University School of Community Health and Policy, Baltimore, MD, United States; 7 University of Southern California Keck School of Medicine, Los Angeles, CA, United States; 8 Tower Health, West Reading, PA, United States; 9 Wake Forest University School of Biomedical Graduate Studies, Winston-Salem, NC, United States; 10 Division of Cardiovascular Medicine, Penn Presbyterian Medical Center, Philadelphia, PA, United States; 11 Hypertension and Nutrition Core Group of IAPEN INDIA Association for Parenteral and Enteral Nutrition, Pune, Maharashtra, India; 12 Novartis Pharmaceuticals Corporation, East Hanover, NJ, United States; 13 Tulane University School of Medicine, New Orleans, LA, United States

**Keywords:** cardiovascular health (CVH), cardiovascular prevention and rehabilitation, life’s essential 8 (LE8), socioeconomic disparities, socioeconomic status (SES)

## Abstract

Socioeconomic disparities continue to impact cardiovascular outcomes in the United States and clinicians are increasingly faced with navigating barriers for patients of low socioeconomic status (SES). The purpose of this review is to briefly discuss unique challenges in this population and to identify resources and strategies for clinicians to help improve cardiovascular health (CVH) guided by the American Heart Association's Life's Essential 8 (LE8) domains. The review is intended for preventive cardiology clinicians across varying disciplines including but not limited to physicians, nurses, nurse practitioners, physician associates, exercise physiologists and dietitians who are involved in delivering patient care. The rationale for this work is that resources which can assist preventive cardiology clinicians in overcoming socioeconomic disparities exist in a rather fragmented manner which can be challenging to access and time consuming to implement. One practical aim of this review, therefore, is to unify those resources and strategies in a way for clinicians to utilize and reference in day-to-day practice. The LE8 domains provide a structured framework for improving overall cardiovascular health and this review will aid clinicians in understanding and overcoming barriers for patients of low SES incorporating open communication with patients, social support, community resources, and digital tools.

## Approach to literature selection

This review is intended as a perspective review as opposed to a systematic review. The authors conducted a focused literature review on the impact of socioeconomic disparities and low socioeconomic status on each of the eight domains which comprise the Life's Essential 8. The review was conducted using the search databases PubMed, Scopus, and Google Scholar with a search timeframe from January 1, 2024 to March 1, 2026. Landmark published papers or primary research from prior to January 1, 2024 deemed highly relevant to the topic were also selected by the authors. The manuscript was collaboratively reviewed and edited with approval by all contributing authors prior to submission.

## 1 Background

Cardiovascular disease (CVD) remains the leading cause of death in the United States since 1921 (1), with atherosclerotic cardiovascular disease (ASCVD) contributing to most of the morbidity and mortality in the United States. Public health and professional organizations are working to improve cardiovascular health (CVH) across the lifespan while navigating the challenges presented by social determinants of health (SDOH)-the conditions in which individuals live, learn, work and socialize (2). Socioeconomic status (SES) is influenced by education, employment, and income. Key domains to improve and maintain CVH are outlined in the American Heart Association's (AHA) Life's Essential 8 (LE8) framework and underscore the necessity for interdisciplinary collaboration among healthcare clinicians to create a comprehensive and personalized care model to address individual needs (3). Most individuals who develop ASCVD have at least one major modifiable risk factor and few individuals have optimal control of all risk factors (4). Despite the overall decline in cardiovascular mortality in the United States, the rate of this decrease has slowed recently. Social factors, coupled with the rising prevalence of diabetes and obesity may be contributing to this deceleration and to poor outcomes (5). SDOH and socioeconomic disparities have led to persistent gaps in screening, insurance coverage and access to care, risk factor control, and cardiovascular outcomes (6–9). For example, removing the cost burden of coronary artery calcium scoring has been shown to lead to increased uptake by patients-sometimes leading to risk re-classification and changes in treatment recommendations and medication eligibility (10).

Clinical tools exist, for example the AHA's PREVENT risk calculator (Predicting Risk of Cardiovascular Disease Events), offer clinicians the ability to incorporate a ZIP code to calculate the social deprivation index as part of a patient's ASCVD and global CVD risk assessment (11, 12). This index includes a geographically-based measure of education, employment, and household characteristics, and represents a shift from previous iterations of risk calculators that incorporate race or ethnic background (11, 12). Household income, in particular, is a major social factor in SES and there is a greater prevalence of cardiovascular risk factors and CVD among low-income households (13, 14).

Incorporating early prevention strategies and reducing the impact of healthcare disparities is of the utmost importance. Research shows that among patients who have established CVD, lower SES increases the risk of recurrent CVD events independent of risk factors (15).

**Figure 1:**
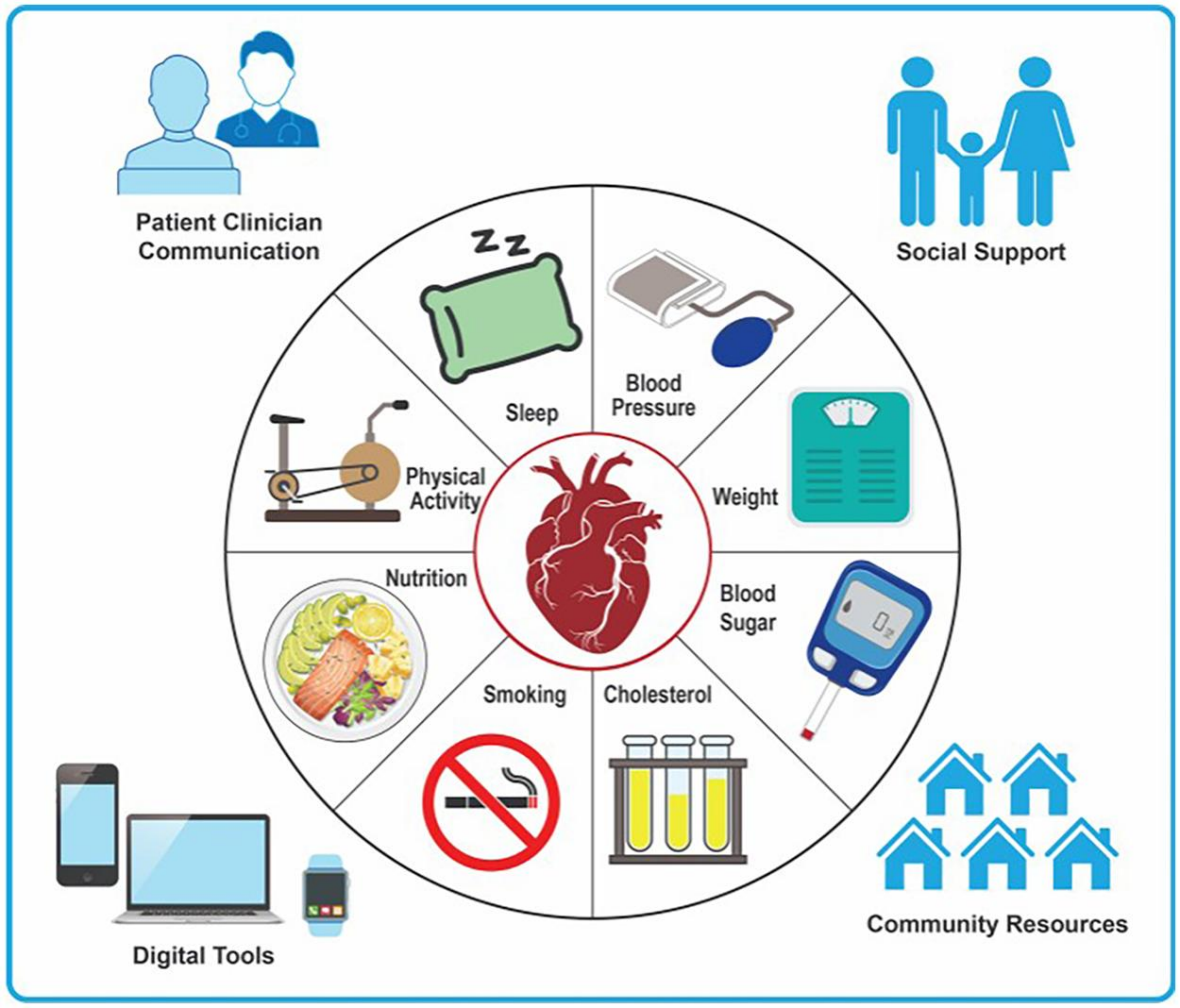
Framework for clinicians facing the impact of socioeconomic disparities on the life's essential 8 (LE8) domains for cardiovascular health.

A challenge faced by preventive cardiology clinicians today is how to reconcile short visit times and prolonged visit intervals with incorporating strategies for overcoming socioeconomic hurdles on an individual patient level. This review highlights how socioeconomic barriers continue to influence the health behaviors and health factors outlined within the AHA LE8 framework while also offering resources and practical strategies for how clinicians can troubleshoot these care gaps within their own clinical capacity. Though the impact of socioeconomic disparities on cardiovascular health extends globally, this work will specifically focus on its impact in the United States (16, 17). We propose a multifaceted approach which includes open communication between clinicians and patients, emphasizes the importance of social support and community resources, and explores the availability and utilization of digital tools (Figure 1).

### 1.1 Eat better

Over 47 million people in the United States live in food-insecure households, defined by the lack of dependable access to the type and amount of food needed for each member of a household to lead an active and healthy life (18–20). As food security status worsens, there is a statistically significant increase in the prevalence of cardiovascular disease (21–23).

As clinicians, we frequently advocate for a healthy eating pattern that includes an abundance of fruits and vegetables, whole grains, lean protein with an emphasis on plant protein, limited intake of alcohol, and the avoidance of saturated fats and added sugar (24). Studies have shown that adoption of a Mediterranean-style diet is associated with a lower incidence in cardiovascular disease in addition to lower all-cause mortality and can be a strategy for patients from any cultural background (25–27). Clinicians should start by assessing a patient's current cultural diet and aligning the health-promoting elements of the Mediterranean diet with foods familiar to the culture and that patients find palatable (28). A registered dietitian can be a tremendous resource in this effort as well.

A recent review suggested that the cost of the Mediterranean-style diet may present a barrier to access and adherence for some individuals despite geographic location (29). Thus, due to financial constraints, individuals living in food insecure households may have lower quality diets than those living with food security (21, 30–33).

To ensure that patients living in food-insecure households have the same opportunity to improve CVH as those living in food-secure households, clinicians need to proactively inquire about food accessibility as well as be prepared to provide appropriate resources for patients to access and consume healthy meals. Standardized screening for food insecurity—along with screening for other SDOH– can help to overcome this stigma, identify vulnerable individuals, and reduce the prevalence of food insecurity and its impact on the community. This could improve outcomes and reduce healthcare costs (19, 34). Though multiple professional societies recommend utilization of a food insecurity screening tool, the United States Preventative Services Task Force (USPSTF) recently concluded there is insufficient evidence to assess the balance of harm and benefit of screening for food insecurity in the primary care setting (19, 34–36). It may still be helpful to incorporate screening into patient care. Selected tools that are brief may be incorporated into routine healthcare visits. For example, the Hunger Vital Signs™ is a validated two-question screening tool, often embedded into the Electronic Medical Record (EMR), that is used to assess food security status in clinical practice and can help foster discussion with patients when appropriate (34).

When food insecurity for a patient is identified, appropriate referrals should be offered with resources to connect individuals to healthy food options. If available, a referral to social work, case management, or a community health worker is an appropriate first step to help patients with sustainable resources. Providing resources about local food banks can help with immediate access to food. Local food bank information can be found through (FeedingAmerica.org) (37). For individuals who qualify for the Supplemental Nutritional Assistance Program (SNAP), resources to help determine eligibility and how to apply should be easily accessible, and for those eligible for SNAP benefits, they may access online ordering to reduce barriers for those with limited transportation, mobility restrictions, or fewer convenient options for utilizing the program benefits (38).

In addition to facilitating improved access to healthy foods, nutritional education for a heart-healthy diet should be incorporated. Whenever possible, referrals to nutrition therapy by a registered dietitian can offer the patient tailored nutrition education with heart-healthy foods that fit within their financial budget. Should a clinical dietetics department be unavailable, clinicians can utilize educational resources available on the U.S. Department of Agriculture's Nutrition, MyPlate, and SNAP Education websites (37–39). Finally, the American Society for Preventive Cardiology's 2022 *Practical, Evidence-Based Approaches to Nutritional Modifications to Reduce Atherosclerotic Cardiovascular Disease* Clinical Practice Statement is a comprehensive resource for clinicians to understand the role of a heart-healthy diet in improving CVH, along with clinical guidance for working with patients (40).

### 1.2 Be more active

Consistent exercise reduces ASCVD risk in the short, middle, and long-term in a dose-dependent manner (41–43). Current guidelines recommend that all adults regularly participate in aerobic-based exercise training as part of lifestyle modification for managing heart health (41–44). Participating in at least 150 min weekly of moderate-intensity aerobic-based exercise training is associated with reducing ASCVD risk and improving control of individual risk factors, including the lowering of blood pressure, cholesterol, weight, and glycated hemoglobin (HgBA1c) (45–47). Accomplishing weekly training volumes that exceed minimum recommendations by at least two-fold further strengthen the cardioprotective effects of aerobic-based exercise at any age (41–43).

When considering the impact SDOH and SES can have on limiting choices, not only for exercise, but also for leisure time physical activities, neighborhood characteristics can play an important role. Patients who reside in areas of low SES tend to experience limited opportunities for accessing parks, dedicated sidewalks/walking paths, and natural green spaces. These patients are more likely to live in neighborhoods with higher rates of violence, increased density of unhealthy food retailers, and overall lack of safe space to promote physical activity (48). By contrast, proximity to parks has been shown to improve physical activity time in older adults and a higher number of walkable destinations and physical/recreational facilities within 5 km of the home has been linked to improved cardiovascular health among older adults (49, 50). Also, the presence of social support of friends and family increases the likelihood of engaging in regular exercise and physical activity (51).

Considering these factors together is essential because regular participation in leisure-time physical activities inversely correlates with ASCVD risk, even among those who do not regularly participate in any aerobic-based exercise training (52, 53). Given women in low-income households are more likely than men to serve the at-home role of primary caregiver to younger and/or older aged family members, it is also relevant that the inverse association between leisure-time physical activity and ASCVD risk may be stronger in women than men (52). It follows that disadvantages experienced by patients that limit exercise and physical activity participation are not typically isolated to the physical indoor and outdoor living space, but coexist with overlapping socioeconomic factors such as financial and employment insecurity, unreliable or lack of private/public transportation, limited or no available affordable/free local gyms, primary caregiver responsibilities, etc.

Identifying the impact of SDOH and SES should be an essential part of the initial clinical interview during which patients are asked to describe their current types and levels of daily physical activities, including any formal exercise participation. For patients who experience barriers to joining a gym, purchasing home equipment, walking in the neighborhood, etc., as modes of exercise, it is important to emphasize that aerobic-based exercise training can still be effective via approaches that focus on available resources within the immediate home space. All components of a personalized exercise prescription (i.e., frequency, intensity, time, type, and progression) can be developed for a program focusing on moderate intensity (i.e., breathing is elevated, but talking is comfortable) (54). Walking hallways of the home or walking around the space surrounding the dining room table can be useful strategies. Other indoor options available year-round could include the use of aisleways at local shopping centers during non-peak business hours, or even the physical space at the workplace during lunch breaks or before/after shifts. Additionally, persistent efforts to improve physical activity resources in local neighborhoods is an important cardiovascular prevention strategy from a public health perspective.

While primary prevention of cardiovascular events is the focus of this review, clinicians caring for patients who have experienced a cardiac event or have a chronic cardiac condition should assess eligibility for cardiac rehabilitation (CR). Unfortunately, many eligible patients do not enroll in CR due in part to socioeconomic factors such as limited transportation availability and out-of-pocket costs (55, 56). Early case management involvement has been shown to improve cardiac rehabilitation adherence in patients of lower SES (55, 56). Patients should be educated on available resources such as financial counselors and transportation services associated with the medical institution and/or insurance provider to assist with limiting the impact of these socioeconomic barriers.

### 1.3 Quit tobacco

Cigarette smoking has long been associated with an increased risk of coronary artery disease and stroke as it is associated with a more atherogenic lipid profile, chronic inflammatory state, increased platelet activity and aggregation, and endothelial dysfunction (57, 58). One systematic review found that half of peripheral artery disease cases are due to smoking, and 80% of patients with peripheral artery disease are current or former smokers (59). Recently, however, through national and local efforts, smoking rates have decreased over the past few decades, possibly due to rising costs in cigarettes (60). Since 1965, long-term smoking rates have dropped from 42.6% to 11.8% as of 2022 and cigarette consumption continues to decrease among the 28.8 million current U.S. smokers (61, 62). Perhaps as a substitute, though, more Americans have switched to other forms of nicotine, as the use of e-cigarettes and vapes is more prevalent, with 15 million people using e-cigarettes as of 2022 (61, 62).

Cigarette use is disproportionately more common among individuals of low SES, low educational levels, and those with psychiatric comorbidities, substance use disorders, and those with disabilities (63–65). As the average cost of cigarettes in the United States is approximately $10 per pack, the financial burden imposed by ongoing nicotine use may impair efforts to improve other domains of cardiovascular health (66). Additionally, cigarettes smoked per day is highest in unemployed individuals, and a higher degree of nicotine dependence was associated with a lower household income (63). There are also racial disparities in tobacco use among adolescents. Observed decline in tobacco smoking among non-Hispanic White high school and middle school students has been significantly greater than the decline of tobacco smoking in non-Hispanic Black and Hispanic/Latino students, suggesting the reduction in adolescent tobacco use has not equitably reached communities of color (64). Developing partnerships with community-based organizations with a greater focus on tobacco control is just one recommended avenue for addressing the disparity in tobacco control in the United States (65).

While the CDC reports that most adults want to quit smoking and half of current smokers report cessation attempts in the past year, less than 1 in 10 adults who quit smoking succeed in smoking abstinence 1 year later (61, 62). This may be attributed to adults who smoke not getting advice, assistance, and treatments for tobacco cessation that would benefit them long-term. Brief advice (less than 3 min) from a health professional improves the chance of quitting successfully and is highly cost-effective (61, 67). Clinicians can incorporate evidence-based treatment strategies in counseling, such as the Tobacco Cessation Brief Clinical Intervention Protocol, which provides a pathway for clinicians based on resources and timeframe. Using a combination of counseling, replacement therapy, and medication confers a better chance of success for smoking cessation (61, 67). Additionally, best practices and case studies are provided in the Million Hearts campaign resources, which can be applied to small and larger healthcare systems and leverage existing tools in electronic medical record databases (68). Clinicians may also find more culturally-targeted information and cessation strategies through the American Lung Association's (ALA) Empower Your Community documents addressing strategies in Black, Hispanic, Indigenous, female, and youth communities (69). Clinicians should refer to available evidence-based resources for cessation counseling and medical therapy based on insurance coverage, as presented in ALA's online guide (70).

Finally, mobile applications that incorporate behavioral support and text message-based coaching have shown promise in promoting tobacco cessation as well. One study found that smart phone applications utilizing acceptance and commitment therapy had a 1.49-fold higher rate of quitting than the conventional application teaching avoidance of smoking triggers (71). Many of these applications are free, such as quitSTART, developed by the National Cancer Institute, which offers personalized treatment plans and provides real-time support (72). Other programs, such as the text-based program Text2Quit have been shown to increase cessation attempts significantly when compared to standard intervention (73). While resources are available for patients at low or no cost to quit tobacco, clinicians must continue to openly communicate with patients to prioritize tobacco cessation to improve CVH outcomes.

### 1.4 Get healthy sleep

Research shows that there is a disparity in sleep duration and sleep quality and it is strongly associated with lower SES and race (74, 75). Short sleep duration, defined as less than 7 h per night, is linked to an increased risk of cardiovascular disease and stroke (76). Chronic sleep deprivation contributes to hypertension, elevated inflammation, and atherosclerosis, largely due to abnormal activation of the sympathetic nervous system (77–79). Additionally, chronic sleep deprivation is associated with a higher risk of type 2 diabetes and obesity, which collectively raise the likelihood of cardiovascular events (76, 78). Among those suffering from insomnia, there is a high prevalence of CVD and a connection with cardiac rhythm disturbances like atrial fibrillation (80).

Individuals who work shifts, reside in low SES neighborhoods, or have experienced childhood adversity are more likely to suffer from sleep-related disorders, sometimes due to lower physical activity, anxiety, depression symptoms, and helplessness (81–83). Working individuals of lower SES are particularly impacted, as these individuals may get less sleep due to demanding schedules, the need to balance work and leisure, and challenging living conditions—such as crowded or unsafe housing, noise, and extreme temperatures. Furthermore, individuals of lower SES are more likely to work irregular shifts, which can disrupt circadian rhythm and worsen sleep quality (81, 83, 84).

Among patients with obstructive sleep apnea (OSA), low SES is an independent risk factor for CVD (85). Treatment adherence for OSA has been shown to be lower among individuals in lower SES neighborhoods. Low-income adults are also more likely to forego OSA treatment, with 29% citing cost as the primary reason for this decision (81).

A recent scientific statement from the AHA recognizes that sleep health represents a multidimensional construct beyond just sleep duration (sleep duration, continuity timing, regularity, daytime functioning, architecture and absence of sleep disorders) as it relates to impact on cardiometabolic health (86). The statement, however, acknowledges that guidance on multidimensional sleep health cannot be made without further evidence-based study of its impact on CVH. There is data, though, that extending sleep duration alone can improve cardiometabolic outcomes (87).

Asking patients open ended questions such as “How is your sleep?” and simply recording the responses within the chart to be shared with other involved clinicians can provide information on different dimensions of patient sleep health. RU-SATED is a questionnaire tool that can be implemented in the clinical setting to inform clinicians on multidimensional sleep health (86, 88).

Strategies for improving sleep can be accessible for patients of all SES levels and should be discussed during visits. Several non-pharmacological interventions are available to improve sleep, particularly for patients with ASCVD risk factors. These include environmental modifications (e.g., using earplugs, eye masks, maintaining a consistent wake-up time, creating a cool/dark room, enjoying a relaxing bath, following a bedtime routine, and avoiding caffeine, heavy meals, alcohol, tobacco, and blue light exposure), relaxation techniques, and self-care practices (e.g., deep breathing exercises, physical activity), all of which have shown significant positive effects on self-reported sleep quality (89). A meta-analysis found that music-assisted relaxation, using either patient-preferred music or music specifically composed for relaxation or sleep promotion, provides statistically significant improvements in sleep quality without adverse side effects. Many people find slow rhythm music, especially without a heavy beat, to be particularly relaxing. This approach is a cost-effective, widely accessible resource that requires no intensive training (90). Several studies have demonstrated that mind-body exercises, such as tai chi and yoga, result in substantial improvements in sleep quality, especially for older adults (91). A referral for cognitive behavior therapy should be considered for patients with insomnia as the psychologic treatment of choice (92). Additional targeted interventions to improve multidimensional sleep health in the clinical and community settings are certainly needed, especially for patients of lower SES.

### 1.5 Manage weight

Weight management is a critical factor in overall health, particularly for preventing chronic conditions like obesity and ASCVD (93). However, the ability to maintain a healthy weight is not only dependent on individual behavior but also deeply influenced by socioeconomic factors such as income, education, occupation, access to healthcare, and cultural norms. Disparities in these factors create systemic barriers, disproportionately affecting low-income and marginalized populations, and exacerbating health inequities in the United States. High-income households often have access to nutritious foods, such as fresh produce and lean proteins, while low-income populations face “food deserts” or “food swamps,” characterized by limited availability or an overabundance of unhealthy, calorie-dense foods. Studies highlight the high cost of nutritious diets as a major barrier for low-income families, forcing reliance on processed and cheaper alternatives that contribute to weight gain and obesity (30, 94, 95). Education also influences health literacy of proper nutrition and exercise. Employment conditions play a role in weight management. Low-income jobs can involve long hours, physical fatigue, or shift work, leaving little time for exercise or healthy meal preparation. Occupational stress further exacerbates unhealthy coping mechanisms, such as over-eating, contributing to obesity. In contrast, higher-income workers may benefit from flexible schedules and supportive work environments that facilitate healthy lifestyle choices. Studies have shown that there is a positive linear association between body mass index (BMI) and night shift vs. day shift schedule, and manual labor jobs vs. management or professional work (96). Lastly, chronic stress and financial insecurity seen in low-income populations could impact appetite regulation and contribute to weight gain and obesity (78, 96).

Access to healthcare significantly impacts weight management. Higher-income individuals are more likely to afford health insurance and weight management resources, such as consultation with registered dieticians, exercise physiologists, medications, or bariatric surgery. For example, Glucagon-like-Peptide-1 receptor agonists (GLP-1 RA) medications have shown effectiveness in promoting weight loss in addition to treatment of type 2 diabetes, but their high cost can render them inaccessible to low-income populations, and socioeconomic disparities may therefore restrict their uptake (97, 98). Among patients with type 2 diabetes, there are even racial disparities in prescription of GLP- RAs which impact outcomes (99). In a cohort of patients with type 2 diabetes and ASCVD, lower rates of GLP-1 RA use were found among Asian, Black, and Hispanic patients (98). GLP-1 RAs have become the pharmacologic cornerstone of obesity treatment and ensuring equitable access and reducing costs should be a priority from a public health perspective. Clinicians may look to manufacturer-assisted support or direct to consumer platforms for patients for these costly, yet clearly effective medications. Recently, there have been ongoing government negotiations in this arena and the cost of this effective class of medications is already starting to decrease.

Sustaining long-term weight management through dietary and lifestyle modification remains challenging, but AI-enabled coaching platforms offer potential to personalize recommendations. Research has found that the majority of individuals reported a weight reduction while using a mobile weight loss application, and mobile health applications could help obese and overweight individuals achieve a 5% reduction in weight over a short period of time (100, 101). Integrating remote monitoring with usual care in patients with type 2 diabetes showed improvements in weight, HbA1C while reducing the number of diabetes medications (102).

Finally, cultural attitudes toward diet and body size can influence weight management efforts. In some communities, traditional diets and perceptions of body size may conflict with clinician recommendations, so clinicians must approach these health-focused conversations with open communication and in a culturally sensitive manner.

### 1.6 Control cholesterol

Serum cholesterol assessment in the LE8 has transitioned from total cholesterol to non-HDL cholesterol, in part because non-HDL levels can be measured in non-fasting state, are accurate in all patients, and better reflect the presence of all atherogenic lipoproteins (3). No consistent relationship between lower SES and poorer cholesterol control has been demonstrated in the United States. In an analysis of serial National Health and Nutrition Examination Surveys (NHANES) between 1971 and 2002, the prevalence of high cholesterol (defined as a total cholesterol >240 mg/dL) decreased across all quartiles of income; however, the magnitude of decrease was greatest in those with higher incomes (16% in the highest income quartile vs. 10% in the lowest income quartile) (103). No differences were observed across education status. In an updated NHANES analysis spanning 1999–2018 no consistent differences were observed for total cholesterol levels and changes in total cholesterol over time across income or education (104).

Dietary sources high in saturated fat or trans-fat can significantly increase cholesterol levels and these include fatty meats (like red meat, bacon, sausage), full-fat dairy products, processed meats, fried foods, baked goods, and tropical oils. Though these foods are often less expensive, there is no clear evidence that lower SES is associated with a higher consumption of saturated or trans-fat foods. A systematic review of over 30 studies from low- and middle-income countries found that individuals with high SES consumed more dietary cholesterol and saturated fat compared to those with lower SES (83). Similarly, using serial data from NHANES surveys, while saturated fat intake did not differ at baseline (1999) according to education level, those in the highest education category had the greatest increase in saturated fat intake over time and, by study end (2016), total intake levels in this group were significantly higher compared to those in the lowest education category (105).

Research, however, has consistently shown a strong correlation between lower SES and reduced access to lipid lowering therapy (LLT), including both statin and non-statin options. Lower income, lower educational attainment, and lack of adequate health insurance have all been associated with limited access to LLT (106–109). Any LLT use in middle-aged individuals is nearly three times higher in those with health insurance compared to those without (23.9% vs. 8.1%) (107). Amongst those with insurance, higher income is associated with a greater likelihood of being prescribed statin therapy and adhering to LLT (106, 108, 109). These differences are perhaps most prominent when considering the growing number of novel lipid-lowering therapeutics where no generic version is available, the most widely prescribed one being proprotein convertase subtilisin/kexin type 9 inhibitors (PCSK9i). Individuals in the highest income quartile are 30% more likely to initiate PCSK9i than those in the lowest quartile (110). Amongst insured individuals, lower income groups are more likely to have PCSK9i prescriptions rejected. PCSK9i abandonment rates for approved claims were significantly influenced by copay amounts—7.5% for $0 copays to 75% for $350 + copays (111). Risk of cardiovascular events are 15% to 20% higher in individuals with a rejected or abandoned status compared to those with a paid status (112).

NHANES participants with health insurance were more likely to reach LDL-cholesterol goals compared to those without health insurance (21% vs. 10% in 2001–2004), with the gap only widening over time (35% vs. 11% in 2009–2012) (113). This disparity is exacerbated by the fact that the 2013 ACC/AHA guidelines for lipid management, which largely redefined how clinicians should approach stain eligibility, increased the population in whom statin therapy was indicated more in individuals without health insurance (17.6% vs. 15.6% with health insurance) or with no more than a high school education (17.3% vs. 13.0% with a college education) (114). Estimated benefit from statin therapy in over 500,000 UK BioBank participants was much higher in more socioeconomically deprived quintiles. Compared to no statin therapy initiation, those starting and subsequently maintaining statin therapy in their 50's was projected to gain 703 and 360 life years per 1,000 males and females in the most deprived quintile, respectively, compared with 406 and 94 projected life years gained in the least deprived quintile (115).

Targeted interventions to improve LLT access, therefore, represent an effective strategy to help improve cholesterol control. Examples include policy interventions that expand access to affordable health insurance or implement price controls on medications or outreach initiatives to connect individuals in underserved communities with healthcare providers and pharmacies. In a study of predominantly Latino urban areas in large United States cities, neighborhoods with large noncitizen populations had much higher rates of statin nonadherence compared to those with fewer noncitizens; however, disparities were significantly mitigated in areas where counties guarantee healthcare access to all residents, including noncitizens with or without documentation to reside in the US (116).

### 1.7 Manage blood sugar

Diabetes remains one of the leading risk factors for ASCVD, with 38.1 million American adults diagnosed with diabetes and 97.6 million with prediabetes, yet it is still estimated that at least 8.7 million adults have undiagnosed diabetes (117). The disease disproportionately affects non-White and poor persons, and education and household income are inversely proportionate to the likelihood of having diabetes (118). As with persons living with obesity, hypertension and hyperlipidemia, individuals living with diabetes and socioeconomic barriers to optimal health have a higher risk of complications from the disease. There is a higher prevalence of diabetes in food-insecure households, with a notable rise with increasing severity of food insecurity. The association between inadequate glycemic control and food insecurity is multi-faceted: current research proposes a link to poor nutritional quality, increased psychological stress, and resource shifting (e.g., spending money that would otherwise be used for healthy food on medications or rent (31). The higher cost of healthy foods when compared to poorer quality foods continues to present a challenge for patients to adhere to an optimal diabetic diet (21, 22, 119). In addition to the nutritional recommendations described previously, the CDC's National Diabetes Prevention Program (National DPP) provides a stepwise approach for lifestyle coaches to help patients at risk for developing diabetes to not only improve diet and increase activity, but also to find ways to cope with stress (120). Though considered to be a separate SDOH, access to care- specifically the presence or absence of health insurance- is the biggest predictor of whether patients will receive care for their diabetes, irrespective of if they are diagnosed (118). Health insurance is closely linked to employment and income, and for patients with diabetes, the presence and type of health insurance can dictate what type of diabetic care a patient can receive. Specifically, this encompasses screening and diagnosis, counselling, medical therapy and referrals for specialty care for diabetes-associated comorbid conditions.

The Affordable Care Act (ACA) led to reductions in healthcare costs for patients with diabetes, so continuing to help patients find affordable health insurance through state and regional initiatives is helpful for clinicians and their support staff to be aware of. Additionally, the National Institute of Diabetes and Digestive and Kidney Diseases branch of the NIH has a list of resources for clinicians and patients that can help not only identify health care plans that are best for patients with diabetes, but resources for how to pay for diabetic medications and supplies. Clinicians may also find that some patient assistance programs are available for non-generic medications, and the American Diabetes Association has a Community Connection search tool that can help find a variety of resources for patients with diabetes in a specific geographic area. Lastly, for patients who may have difficulty with access based on geography, transportation or other barriers, the CDC created A Guide for Using Telehealth Technologies in Diabetes Self-Management Education and Support and in the National Diabetes Prevention Program Lifestyle Change Program as a resource for health care teams to engage with patients for diabetes management outside of a healthcare setting.

### 1.8 Manage blood pressure

High blood pressure is the most prevalent and modifiable risk factor for developing cardiovascular diseases. Hypertension, defined as blood pressure ≥ 130/80 mmHg or receiving treatment, affects approximately 47% of US adults (121, 122). The CDC reports that counties in the southeastern region of the United States have the highest prevalence of hypertension, and the eating pattern in this region contributes to the disparity in incident hypertension in Black vs. White adults (123). An understanding of barriers to effective hypertension management is essential for clinicians aiming to improve patient outcomes. Notably, research substantiates the correlation between lower SES and elevated hypertension rates (124). A systematic review of economic policies demonstrated that enhancing insurance coverage and healthcare quality improves both medication adherence and blood pressure (BP) control for United States adults with hypertension. However, challenges such as prior authorization and higher copayments can hinder adherence to antihypertensive regimens, as highlighted by four out of eight studies (6). It is imperative that clinicians identify strategies to bolster adherence and overall health outcomes, and the 2025 High Blood Pressure Guidelines emphasize a multidisciplinary, team-based approach with structured follow-up mechanisms to monitor both patient progress and adherence (121). Community leaders should be engaged to implement screening programs for adults in their communities to ensure timely diagnosis and treatment.

Achieving accurate out-of-office BP measurements using self-measurement remains challenging but is essential in managing high blood pressure. Patients should be instructed to use a validated upper-arm, cuff-based device, and clinicians can recommend resources for validated devices such as validatebp.org. Studies also show that mobile applications significantly enhance medication adherence among adults navigating chronic illness (125). Embracing technology, through patient portals and mobile health applications, can substantially improve patient engagement in health behavior monitoring (126, 127). Significant improvements in hypertension control have been demonstrated with home BP monitoring, particularly when combined with digital supervision. A meta-analysis of 11 randomized controlled trials showed significant improvement in both systolic (−3.85 mmHg) and diastolic blood pressure (−2.19 mmHg) with mobile health intervention. In addition, participants with inadequate BP control at enrollment gained more benefits from remote intervention (128). Mobile applications have facilitated the implementation of remote monitoring at a population level, offering a scalable solution for improved BP control.

Clinicians should always counsel patients to optimize non-pharmacologic interventions for lowering blood pressure, including a low sodium and high potassium diet. The Dietary Approaches to Stop Hypertension (DASH) diets focus on fruit and vegetable consumption along with limiting foods high in saturated fat, though it emphasizes sodium restriction as well (129). By leveraging a multidisciplinary, team-based approach, engaging the community, and harnessing technology, healthcare providers can foster improved health outcomes for patients with hypertension.

## 2 Limitations

This review has several limitations. For one, it is written as a narrative review as opposed to a systematic review and so there may be selection bias in the literature that is selected and referenced by the authors. The paper is specifically framed to focus on the impact of socioeconomic disparities in the United States but it cannot be understated that this issue extends globally and different geographic areas may be impacted differently. Also, there is significant heterogeneity in what constitutes socioeconomic indicators. Some of data referenced in this work is observational and it is a challenge to infer causality from observational data. Also, there is significant variability in availability of community and digital resources across health systems and even in different geographic regions of the United States.

## 3 Conclusion

This review highlights the impact that socio-economic disparities and SDOH have on cardiovascular health in the United States and the ongoing barriers facing clinicians and patients in achieving control and optimization of the domains in AHA's LE8. We propose clinicians should systematically identify socioeconomic barriers across these domains, communicate openly with patients using validated screening tools when available, connect patients to community and insurance based resources, and advocate for health system and health policy initiatives that remove larger, structural barriers to optimizing cardiovascular health. Table 1 provides an overview of some of the barriers faced within each LE8 domain and suggested interventions and available resources. Advances in digital tools and increasing adoption of remote patient monitoring, mobile health apps and artificial intelligence assisted risk stratification—both at the population and individual levels do hold potential to enhance access, improve treatment adherence, and optimize cardiovascular risk factors as well. However, we also acknowledge that advances in these technologies also hold potential to create disparity through digital exclusion, low device literacy, as well as device costs. There are also privacy concerns and algorithm bias related to artificial intelligence to consider.

**Table 1:** Clinician resources for SES barries to improve cardiovascular health (CVH) within LE8 domains.

Life's essential 8 domain	SES barrier	Intervention	Resources
Eat better	Food insecurity Nutritional education	Incorporate food insecurity screening during visits; provide resources for SNAP or local food assistance programs Referrals to a registered dietitian/nutritionist; provide patient education	Hunger Vital Sign or Food Insecurity Screening for Adults Screening Tools; http://www.feedingamerica.org; apply for SNAP benefits USDA's MyPlate; SNAP-Ed Nutrition Education Materials
Be more active	Access to safe recreation spaces/activities Limited access to exercise programs and/or equipment Evaluation for cardiac rehabilitation for eligible patients	Consider safe and accessible spaces, such as shopping centers, schools, and community centers Free digital exercise tools and online resources for accessible physical activities Provide referral to cardiac rehabilitation	USDA’s Exercise and Fitness ToolsUSDA’s Exercise and Fitness Tools USDA’s Exercise and Fitness Tools
Quit tobacco	Continued use of tobacco products	Tobacco use screening during visits Provide individualized tobacco cessation counseling and prescribe tobacco cessation therapies when appropriate	NIH Screening Tools and Prevention Tobacco Cessation Brief Clinical Intervention Protocol CDC Clinical Cessation Tools quitSTART mobile app
Get healthy sleep	Poor sleep quality Treatment of obstructive sleep apnea (OSA)	Screen for sleep quality and sleep duration during visits. Refer for sleep testing when appropriate Provide counselling for treatment of OSA and refer to a sleep specialist when indicated. For patients with difficulty accessing CPAP supplies, provide resources to nonprofit organizations supporting affordable access to CPAP supplies	Ru-SATED Questionnaire CMS CPAP Provider Compliance Tips WSCN CPAP Assistance Programs
Manage weight	Cost of weight management medications Access to weight-loss programs	Referral to patient assistance programs for low-cost or no-cost medications Provide evidence-based weight loss materials during office visit.	Novo Nordisk NovoCare Patient Assistance Program Sanofi Patient Connection Lilly Cares CDC-Recognized Family Healthy Weight Programs
Control cholesterol	Cost of lipid-lowering therapies	Optimize evidence-based low-cost lipid-lowering medications and low-cost pharmacy programs Submit tier exception for higher-cost medication	State Rx Plans: National Resource Center for Rx Assistance Medicaid Drug Rebate Program How to request a tier exception (CMS Part D)
Manage blood sugar	Difficulty managing health behaviors impacting diabetes management Cost of diabetes medications and supplies	Referral to diabetic educator. For at-risk patients, refer to the National Diabetes Prevention Program Refer to low-cost or no-cost programs for patients with or without insurance	National Diabetes Prevention Program American Diabetes Association
Manage blood pressure	Home blood pressure monitoring	Provide resources for low-cost or no-cost blood pressure costs (Medicaid, Medicare or other insurance DME),	Target: BP Loaning Out Devices AMA MAP Self-Monitored BP Coverage: Medicaid
